# Assessment of MMP-2/-9 expression by fluorescence endoscopy for evaluation of anastomotic healing in a murine model of anastomotic leakage

**DOI:** 10.1371/journal.pone.0194249

**Published:** 2018-03-22

**Authors:** Philipp-Alexander Neumann, Vanessa Twardy, Felix Becker, Christiane Geyer, Katrin Schwegmann, Annika Mohr, Andreas Faust, Philipp Lenz, Emile Rijcken

**Affiliations:** 1 Department of General and Visceral Surgery, Muenster University Hospital, Muenster, Germany; 2 Department of Radiology, Muenster University Hospital, Muenster, Germany; 3 European Institute for Molecular Imaging, University of Muenster, Muenster, Germany; 4 Department of Palliative Medicine, Muenster University Hospital, Muenster, Germany; University of South Alabama Mitchell Cancer Institute, UNITED STATES

## Abstract

**Background:**

Disturbance of intestinal wound closure leads to insufficient anastomotic healing and is associated with considerable morbidity following colorectal resections. Matrix metalloproteinases (MMPs) play a crucial role in regulation of wound closure. Here fluorescence endoscopy was evaluated for assessment of MMP-2/-9 expression during failed intestinal anastomotic healing.

**Methods:**

Distal colonic anastomoses were performed as a model for disturbed healing in 36 Balb/c mice. Healing was evaluated endoscopically, macroscopically, and histologically after 1, 3 and 5 days. For detection of MMP-2/-9 expression fluorescence endoscopy (FE) was used following *i*.*v*.*-*administration of a Cy5.5-labeled MMP-2/-9 specific tracer. FE was complemented by quantification of the fluorescence signal using the MS-FX PRO Optical Imaging System. An overall leakage score was calculated and correlated with the results of FE.

**Results:**

With increasing incidence of anastomotic leakage from POD1 (17%) to POD5 (83%) the uptake of the MMP tracer gradually increased (signal-to-noise ratio (SNR), POD1: 17.91 ± 1.251 vs. POD3: 30.56 ± 3.03 vs. POD5: 44.8 ± 4.473, P<0.0001). Mice with defective anastomotic healing showed significantly higher uptake compared to non-defective (SNR: 37.37± 3.63 vs. 26.16± 3.635, P = 0.0369). White light endoscopy and FE allowed evaluation of anastomotic healing and visualization of mucosal MMPs *in vivo*. Using FE based detection of MMPs in the anastomosis, an overall positive predictive value of 71.4% and negative predictive value of 66.6% was calculated for detection of anastomotic leakage.

**Conclusion:**

During disturbed anastomotic healing increased expression of MMP-2/-9 was observed in the anastomotic tissue. Fluorescence endoscopy for detection of MMP-2/-9 during the healing process might be a promising tool for early identification of anastomotic leakage.

## Introduction

Anastomotic healing following colorectal resections is a tightly balanced process that is influenced by a plethora of cellular and non-cellular mediators [1]. The final stability of the anastomosis is mainly guaranteed by tension-free technique and undisturbed perfusion of the anastomotic tissue. However the healing process can be influenced by patient specific factors such as the inflammatory condition of the tissue as well as treatment related factors such as immune suppression due to preoperative chemotherapy or radiation [2, 3]. Independent of technical advancements in surgery, anastomotic leakage still occurs in up to 37% of the cases [4–10]. Considering the significant impact on morbidity and mortality associated with its development, anastomotic leakage can be regarded as the major complication following colorectal resections [10, 11]. Furthermore anastomotic complications significantly reduce the quality of life and even the 5-year-survival of patients with colorectal cancer [12]. Clinical presentation of patients can vary from mild signs of inflammation up to advanced septic reaction and ultimately lead to death of the patient [13]. For the surgical treatment timely detection of anastomotic leakage is of major importance for prevention of further damage such as abscess formation, peritonitis and sepsis.

Especially in the early postoperative period and in asymptomatic patients detection of leakage is oftentimes very delicate. Different attempts to define algorithms for detection of anastomotic leakage have been made, but up to date no uniform score has evolved as a gold standard [14–18]. Lately novel techniques have been developed to enhance sensitivity of diagnostic measures. Especially endoscopy has advanced both in detection as well as treatment of anastomotic leakage following colorectal surgery [19]. In the field of imaging techniques, fluorescence based analysis brings the potential to analyze molecular processes during the course of anastomotic healing. On the biochemical level, matrix metalloproteinases (MMPs) play a significant role in reorganization of the extracellular matrix during the remodeling phase of anastomotic healing and overexpression of MMPs has been associated with anastomotic leakage [5, 19, 20]. Recently Faust et al. [21] have developed a fluorescently labeled marker for *in vivo* detection of MMPs, which has also been validated for fluorescence endoscopy to detect early colonic neoplasia but so far has not been used in context of anastomotic healing [22]. Within this study we used fluorescence endoscopy as a novel approach to correlate anastomotic MMP expression with development of anastomotic leakage.

## Materials and methods

### Animal experiments

All animal experiments were conducted according to the protocol approved by the animal care committee of the regional administration of Muenster, Germany (Protocol No. 84–02.04.2014.A241). For the experiments male Balb/c mice of 8–10 weeks and body weight of 18–28g were purchased from Charles River (Kent, England). Male mice were chosen to prevent inhomogeneity during the operation and to rule out hormonal influences on the inflammatory reaction. All mice were kept at the local animal facility one week prior before start of the experiments for acclimatization. Following termination of the experiments mice were sacrificed by cervical dislocation under general anesthesia.

### Operative technique

**Fig 1:**
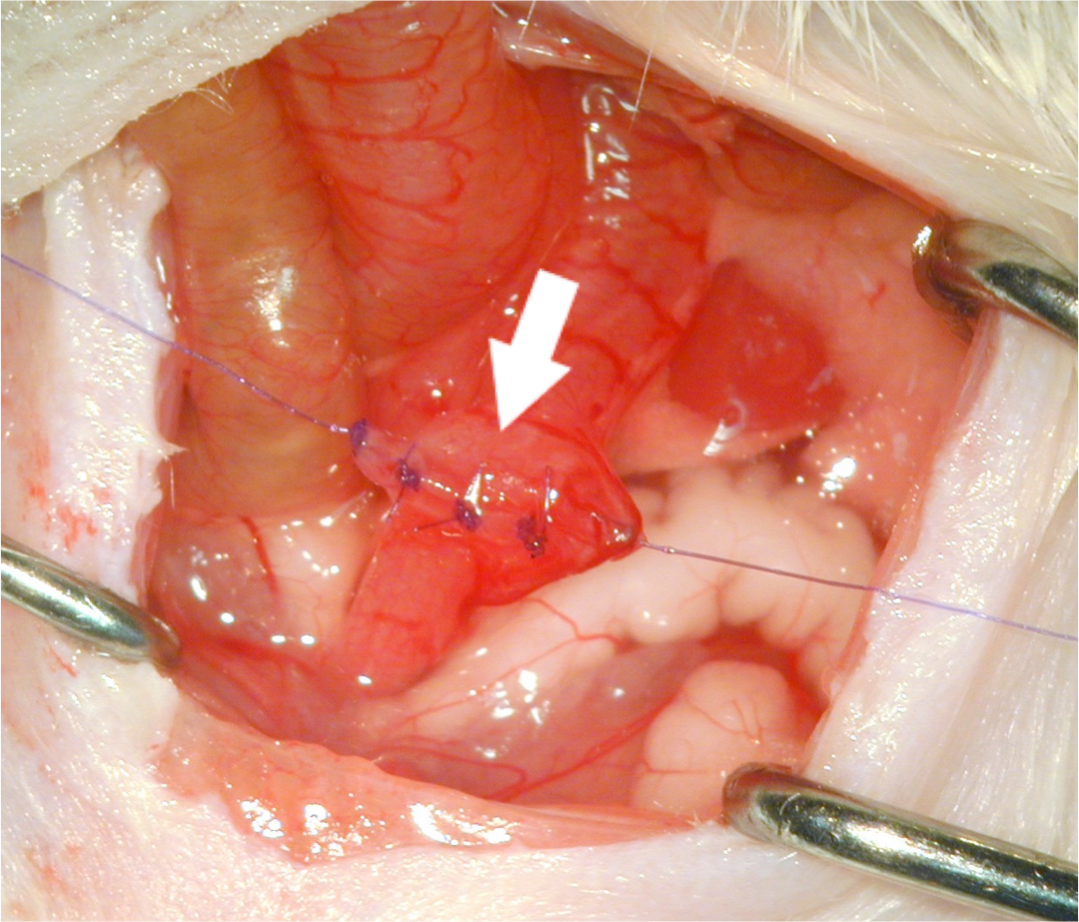
For analysis of anastomotic healing a distal colonic end-end anastomosis was performed. The small bowel was moved to the side and a distal colonic segment at the recto-sigmoid junction was dissected preserving the vascular arcades of the large bowel. A standardized end-to-end anastomosis was performed using 8 single stitches of 7–0 absorbable suture material, each with 4 full thickness single stitches of the anterior and posterior wall.

All surgical procedures were performed in spontaneously breathing animals anaesthetized with isoflurane (Forene, AbbVie Germany) and nitrous oxide (Westfalen AG, Muenster, Germany). Perioperative analgesia was achieved via subcutaneous injection of 2μl Meloxicam per 10g body weight (Metacam, Boehringer Ingelheim Pharma GmbH & Co. KG, Germany). Median laparotomy was performed in supine position. The small bowel was moved to the side and a distal colonic segment at the recto-sigmoid junction was dissected but not resected preserving the vascular arcades of the large bowel. A standardized end-to-end anastomosis was performed using 8 single stitches of 7–0 absorbable suture material (PDS II, Ethicon, Johnson & Johnson Medical GmbH, Norderstedt, Germany). The technique was consistent in all animals, each with 4 full thickness single stitches of the anterior and posterior wall leaving small spaces between the stitches to provoke leakage formation **(Fig 1)**. After recovery from the procedure the animals were removed to their cages where they had free access to regular drinking water and food *ad libitum*.

### Analysis of anastomotic healing

For evaluation of anastomotic healing, the anastomoses were analyzed on postoperative days 1, 3 or 5 respectively. Endoscopic evaluation as well as histologic assessment of the anastomotic tissue was accompanied by macroscopic analysis of the anastomosis *in situ*.

### White light endoscopy for evaluation of the anastomosis

Prior to sacrificing the mice on the respective days of analysis (POD1, 3 and 5), anastomotic healing was evaluated *in vivo* using a murine colonoscope (Coloview Endoscopic System^®^, KARL STORZ GmbH & Co. KG, Tuttlingen, Germany). Endoscopic assessment of the anastomosis was performed under sedation with isoflurane. To quantify the severity of anastomotic leakage, the dehiscence of the anastomosis was measured using the imaging software ImageJ^®^ 1.49 (Rasband, W.S., ImageJ, U. S. National Institutes of Health, Bethesda, Maryland, USA). Dehiscence was defined as the length of the gap between the proximal and distal end of the anastomosis. Furthermore an endoscopic score was developed to assess the healing process of the anastomosis using visible criteria of anastomotic leakage. For this purpose the following parameters were analyzed and scored: 1 point: signs of inflammation without dehiscence, 3 points: dehiscence with signs of bleeding or fibrin formation involving less than 50% of the anastomosis, 6 points: dehiscence with signs of bleeding or fibrin formation involving > 50% of the anastomosis.

### Evaluation of postoperative adhesion formation

Following termination of the postoperative period on POD 1, 3 or 5 respectively, the abdomen was re-opened to intra-abdominally assess the anastomosis *in situ*. Due to development of anastomotic leakage, similar to a covered perforation, adhesions of the surrounding organs to the site of leakage developed. This process of adhesion formation was used as a measure to score anastomotic leakage. The following score was used to quantify adhesion formation (modified from Zühlke et al. [23]): 0 pts, no visible adhesions; 1 point, adhesion could be removed by blunt dissection; 2 points, adhesions needed to be removed by sharp dissection; 4 points, adhesion could not be removed.

### Histologic scoring

For histological scoring of the anastomotic tissue, the anastomosis was lengthwise cut in half and consecutive 4 μm sections of the anastomosis were prepared. Of each anastomosis hematoxylin & eosin (H&E) staining was performed according to regular protocols. The scoring was performed by an experienced pathologist in a blinded fashion. As a measure for anastomotic leakage the severity of the inflammatory response was assessed. In general the anastomotic tissue was analyzed for signs of acute or chronic inflammation (presence of polymorphonuclear leucocytes, lymphocytes and macrophages) as well as remodeling of the tissue. A score was used (adapted from Philips et al. and Attard et al. [11, 24]) to quantify the inflammatory response of the anastomotic tissue: 0 points, no signs of inflammation; 1 point, signs of beginning inflammation; 2 points, signs of active inflammation, 4 points, signs of chronic inflammation. Active inflammation was defined as presence of inflammatory cells such as polymorphonuclear leucocytes as well as lymphocytes. Chronic inflammation was defined as presence of macrophages and scar formation.

### The fluorescently labeled MMP ligand "AF443-Cy 5.5"

For detection of MMP expression in the anastomotic tissue, the fluorescently labeled probe AF443-Cy5.5 was used. It has been designed and initially validated by Faust et al. [21]. In brief, AF443-Cy5.5 is a MMP-2/-9 specific ligand based on a barbiturate analogue structure. The probe was intravenously injected into the tail vein 24h prior to detection. Each animal received 2 nmol of the tracer diluted in 0.1 ml sodium chloride 0.9% (81). To rule out non-specific binding of the tracer at the site of inflammation, an equimolar mixture of an unspecific fluorochrome (Cy3.5-Glycine) and the specific tracer Cy5.5-AF443 was injected in the same mice (n = 9). Cy3.5 and Cy5.5 have a similar chemical structure; co-injection of both fluorophores allows to distinguish the binding of the specific and unspecific tracer without having a pharmacokinetic bias.

### Ex vivo florescence imaging

Detection of the fluorophore *ex vivo* was performed in collaboration with the institute for clinical radiology of the university hospital of Muenster, Germany (TRIC/ translational Research Imaging Center). Following application of the fluorescently labeled tracer 24h prior to analysis, *ex vivo* quantification of the fluorescent signal was performed using the optical imaging system „MS Fx Pro”(Bruker BioSpin MRI GmbH, Ettlingen, Germany). In brief, the intestine carrying the anastomosis was cut lengthwise and analyzed using near-infrared detection as described previously [25]. For anatomical orientation a white light/ gray scale picture was performed and used with the fluorescent signal (Cy5.5: excitation = 630 nm; emission = 700 nm, Cy3.5: excitation = 570 nm; emission = 620 nm). For data analysis the molecular imaging software 7.1.3 (Bruker BioSpin MRI, Ettlingen, Germany) was used. Regions of interest (ROI) were defined around the anastomosis and analyzed compared to background in the distal part of the colon for calculation of the signal-to-noise ratio (SNR) in arbitrary units, as described previously [22, 26].

### Immunohistochemistry

Immunohistochemical detection of MMP-9 was performed as described previously [22, 26]. In brief, paraffin embedded sections of the anastomotic region were made from representative anastomoses of each respective day. The sections were deparaffinized by incubation with xylol (2x 10min) and a series of alcohol solutions of decreasing alcohol concentrations (100, 96, 80, 70% isopropanol, 5 min each). Heat-induced antigen retrieval was performed by incubation in citrate buffer (pH 6) for 30 min. Sections were blocked in 4% goat serum/0.25% Triton X-100/PBS for 30 min. and then incubate overnight at 4°C with an anti-MMP-9 antibody (1:100; Abcam; Cambridge, UK). Isotype-IGG controls were used for proof of specificity (**S1 Fig**). Following washing steps with PBS the slides were incubated with a TRITC-conjugated secondary anti-rabbit antibody (Jackson Immuno Research Laboratories, West Grove, USA). For nuclear staining DAPI was used (Vector Laboratories, Burlingame, CA). The fluorescent images were acquired using the Nikon TE 2000-S Microscope (Nikon, Tokyo, Japan) and the NIS-Elements BR 2.30 imaging software.

### Gelatin zymography

To detect the enzymatic activity of MMPs within the anastomosis, gelatin zymography was performed as previously described [22, 26]. Briefly the anastomotic tissue and non- operated normal murine colon samples as controls, were rapidly frozen in liquid nitrogen and ground using a micro-dismembrator (Sartorius, Göttingen, Germany). The resulting powder was reconstituted in ice-cold NaCl buffer (50 mM Tris/HCl [pH7.5], 75 mM NaCl) containing 1 mM phenylmethylsufonyl fluoride. Samples were centrifuged at 4°C for 20 minutes at 13,000g. Total amounts of protein were determined from the supernatants using a protein assay kit (Bio-Rad, München, Germany). 1 μg of total protein was mixed with 2 x SDS buffer without reducing agent and then added to an 8% SDS gel containing1g/l gelatin. Following electrophoresis the gel was washed using 2.5% Triton X-100 for 30 min at room temperature on a shaking rack. The Zymogram was then incubated overnight at 37°C in a zymogram developing buffer (50mM Tris, 0,2 M NaCl, 5 mM CaCl_2_, 0,02% Brij 35). Subsequently staining with Coomassie Blue R-250 was performed for 60 min. For destaining 45% methanol/10% acetic acid was used. Gelatinase activity appear as light bands in the dark blue background of the stained gel.

### Calculation of the anastomotic leakage score

For quantitative and semi-quantitative comparison of those anastomoses with leakage and those without leakage an anastomotic-leakage-score was defined and mice categorized into two groups (0, mice without leakage; 1, mice with leakage). Leakage was defined as positive results in 3 of the following 4 parameters: endoscopic measures (*dehiscence*, *visible criteria*), macroscopic evaluation (*adhesion formation*) and histological analysis (*inflammation score)*. In case of continuous variables the median of all values was calculated and those values over the median were defined positive for the leakage score. For nominal variables elevated/positive scores were rated as positive for leakage.

### Statistical analysis

All statistical analysis was performed using GraphPad Prism version 7 (GraphPad Software, La Jolla California USA). To present the different variables of the leakage score as well as for presentation of the leakage score in relation to the different groups, contingency tables were used. For quality analysis of the fluorescence endoscopy sensitivity, specificity and the positive predictive as well as negative predictive values were calculated. For calculation of statistical independence of the variables unpaired, two tailed T-tests or Mann Whitney u-tests were performed as appropriate. For analysis of significance of multiple samples ANOVA analysis of variance was used in case of normal distribution or Kruskal-wallis tests were performed for non-normal distribution respectively. A p-value < 0.05 was considered significant.

## Results

### Validation of the model for analysis of anastomotic leakage

**Fig 2:**
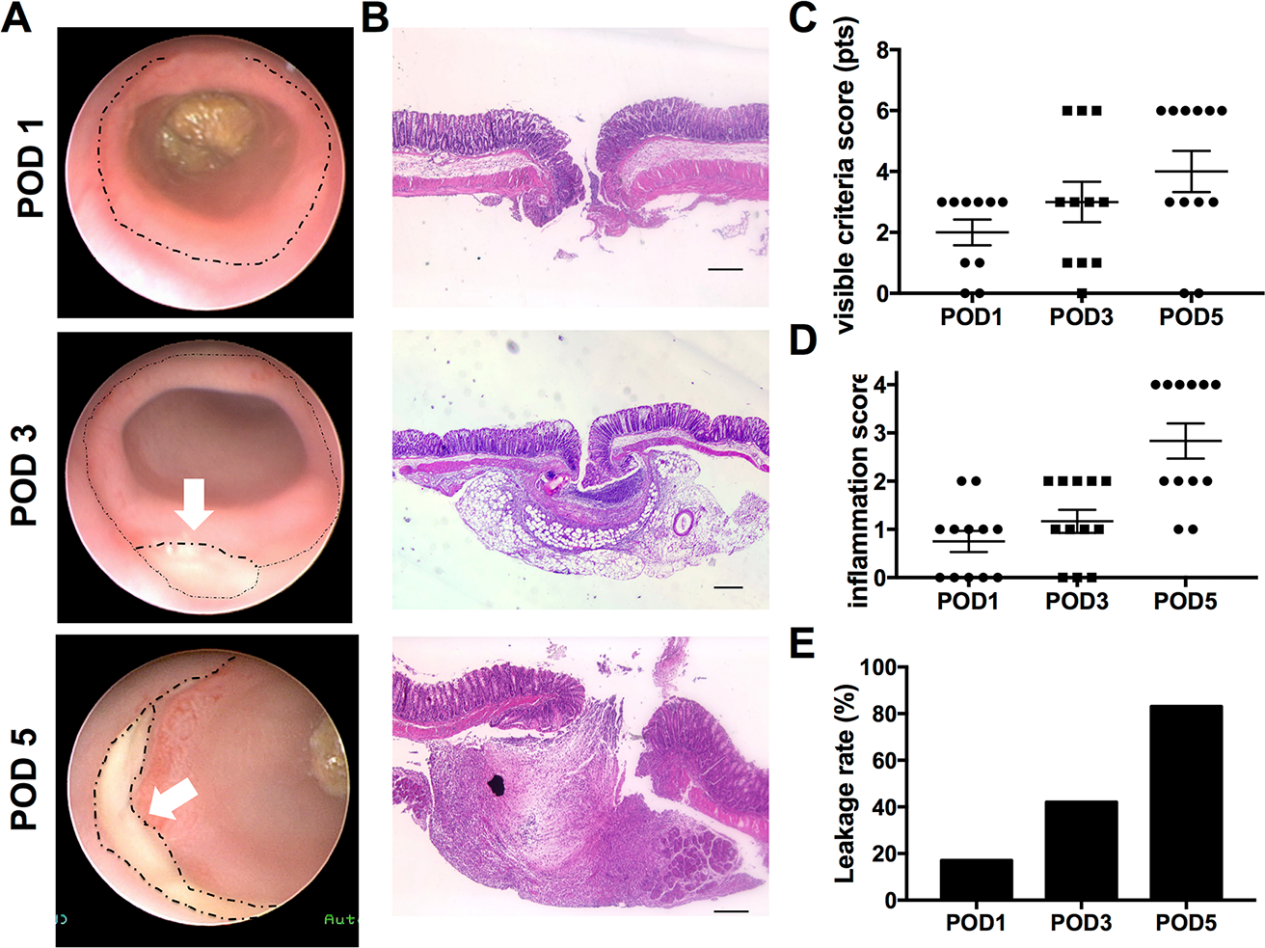
The chosen model serves as a valid tool for analysis of anastomotic leakage. **(A)** Representative images of endoscopic evaluation of anastomotic healing, anastomosis marked by dotted line, arrows indicate dehiscence. **(B)** Representative images of histological sections of the anastomoses on the respective postoperative days. Scale bar 150 μm **(C)** Endoscopic scoring of visual signs of leakage revealed significantly higher scores on POD5 compared to POD1 (p = 0.0428). **(D)** Histological inflammation scores were significantly higher with pronounced anastomotic inflammation on the later postoperative days (p = 0.0006). **(E)** An anastomotic leakage score was calculated taking all parameters into account. As shown in the graph, leakage rates significantly increased on the later postoperative days (p = 0.0047).

For evaluation of MMP expression during anastomotic leakage, first an anastomotic leakage model was established and validated using endoscopic, macroscopic and histologic parameters of analysis (**Figs 1 and 2**).

Prior to termination of the experiment on the respective days, white light endoscopy was employed to analyze anastomotic healing and calculate the leakage score based on endoscopically visible criteria as shown in **Fig 2A**. On POD1 and POD3 a total of 3 mice had to be excluded because of technical impossibility to endoscopically analyze the mice due to obstruction by fecal content that could not be removed. Mean values of the score increased from POD1 to POD5 (POD1: 2 ± 0.42 pts; POD3: 3 ± 0.66 pts; POD5: 4 ± 0.67 pts) with a significant difference between POD1 and POD5 (p = 0.0428) (**Fig 2**). In support of the model, on POD1 none of the anastomoses reached a top score of 6 pts, whereas on POD5 50% of the anastomoses were scored with maximum points indicating increasing leakage rates. For further endoscopic quantification of anastomotic leakage, the width of the anastomotic line (dehiscence) was measured as the maximal distance between the proximal and distal wound edge (**Fig 2A)**. Comparison of mean values revealed increasing dehiscence on the respective postoperative days (POD1: 76.6 ± 29.0μm; POD3: 164.5 ± 48.24μm; POD5: 253.2 ± 53.49μm) with significantly higher values on POD5 compared to POD1 (p = 0.0127).

Due to the anastomotic leak, peri-anastomotic adhesions of the intra-abdominal organs developed analogous to development of a covered perforation. Therefore manifestation of adhesion formation was used as a surrogate marker for anastomotic leakage and quantitatively compared on the different postoperative days. Overall explorative analysis of the data showed a significant difference between the days (p = 0.0002). Analysis of the respective days revealed significantly increasing adhesion formation as a sign of increasing intra-abdominal inflammation with increasing postoperative days (POD1: 1.33 ± 0.41pts; POD3: 2.17 ± 0.35pts; POD5: 3.83 ± 0.17pts). Performance of Mann Whitney U-test showed significant differences between POD1 and POD5 (p<0.0002) as well as POD3 and POD5 (p = 0.0017).

To evaluate whether the macroscopic findings were reflected by histological evidence of anastomotic leakage H&E staining of the anastomotic regions were performed and scored (**Fig 2B**). Comparison of the inflammatory score (range, 0–4) of the anastomoses showed increasing values throughout the postoperative period (POD1: 0.75 ± 0.21pts; POD3: 1.17 ± 0.25pts; POD5: 2.83 ± 0.36pts). In the explorative statistical analysis a significant overall difference was calculated (p = 0.0006). Post-hoc analysis revealed significantly higher histological inflammation scores on the later postoperative days indicating increasing inflammatory reaction over time (POD1 *vs* POD5 (p = 0.0003) and POD3 *vs* POD5 (p = 0.0048)).

To quantitatively compare MMP expression in the group of anastomoses with leakage to those without leakage, all anastomoses have been grouped in a binary system as either insufficiently or sufficiently healed. Insufficient healing was defined taking as 3 out of 4 positive values of the above parameters. As depicted in **Fig 2E,** in comparison of the number of insufficient anastomoses within the different days, there was a higher leakage rate with increasing postoperative days (n, POD1: 17%, POD3: 45.5%, POD5: 83%). Altogether the evaluated parameters as well as the overall leakage score revealed an increase in anastomotic leakage over time. Using the leakage score the model allows comparison of insufficiently vs. sufficiently healed anastomoses.

### Analysis of MMP expression during development of anastomotic leakage

**Fig 3:**
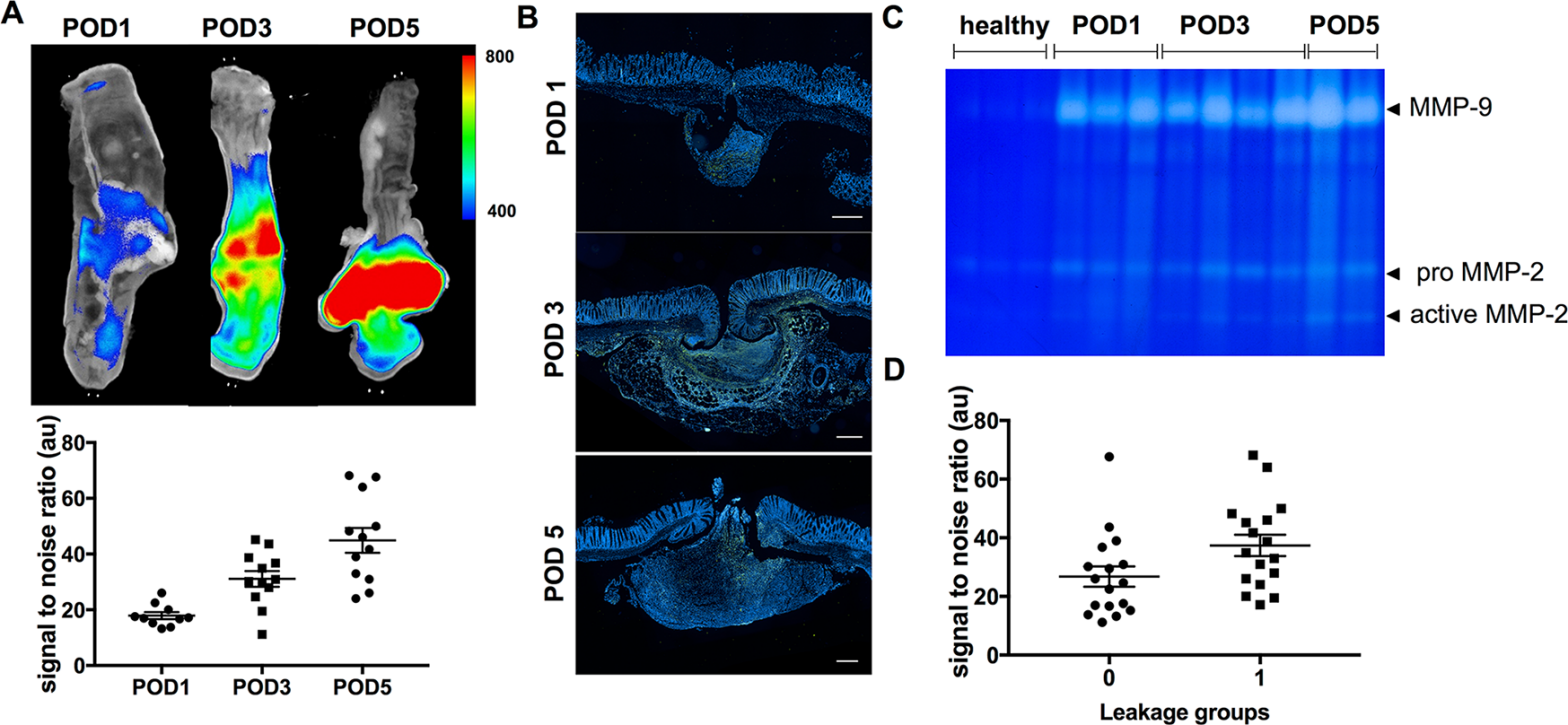
MMP expression increases during anastomotic healing and correlates with anastomotic leakage. **(A)** The photoprobe *AF443-Cy5*.*5* was injected 24h prior to analysis and was detected with significantly increased intensity on the later postoperative days (SNR, POD5 *vs*. POD1, p <0.0001). **(B)** Using immunostaining MMP-9 expression can be localized within the anastomotic tissue as well as the perianastomotic inflammatory exudate with increased expression beginning on POD3. Scale bar 150 μm. **(C)** Immunostaining was corroborated using gelatin zymography. As shown in the gel, elevated expression and activity of MMP-2 and -9 can be detected beginning on POD3. **(D)** In comparison within the leakage groups (0 = no leakage; 1 = leakage) the leakage group showed significantly higher uptake of the tracer in *ex vivo* analysis (SNR: vs. 37.37± 3.63 vs. 26.16 ± 3.635, p = 0.0369).

After verification of the anastomotic leakage model, we sought to investigate the MMP expression pattern within the anastomosis over the healing period. We therefore investigated the potential of the fluorescently labeled probe for *ex vivo* detection of MMP within the anastomotic tissue. Thus the photoprobe was injected 24h prior to analysis and the signal-to-noise ratio (SNR) around the anastomoses was calculated. Significant increase of SNR could be detected from POD 1 to POD 5 with pronounced detection of the signal on POD5. As shown in **Fig 3A**, the detected signal-to-noise ratio revealed significantly increasing mean values over time (POD 1: 17.91 ± 1.251 vs. POD3: 30.56 ± 3.03 vs. POD5: 44.8 ± 4.473, one-way ANOVA, p<0.0001).

To corroborate the fluorescence measurements with localization of MMP expression within in the anastomosis, immunohistochemistry was performed. As shown in **Fig 3B**, MMP expression was detected with peak expression in the anastomotic area. Most specifically increasing signs of MMP expression could be detected from POD1 to POD5. For semi-quantitative analysis and enzyme-activity-measurements, gelatin zymography was performed. As shown in **Fig 3C** an increase in enzyme activity beginning on POD3 could be detected, reflecting increase in MMP expression and enzymatic activity over time. We subsequently wanted to correlate MMP expression with the development of anastomotic leakage over time. Thus the SNR values of the anastomotic region were compared within the two leakage groups (0, no leakage; 1, leakage). Here a significantly higher uptake of the tracer could be detected in the leakage group (SNR: 26.16 ± 3.65 vs. 37.37± 3.63, P = 0.0369) **(Fig 3D**).

**Fig 4:**
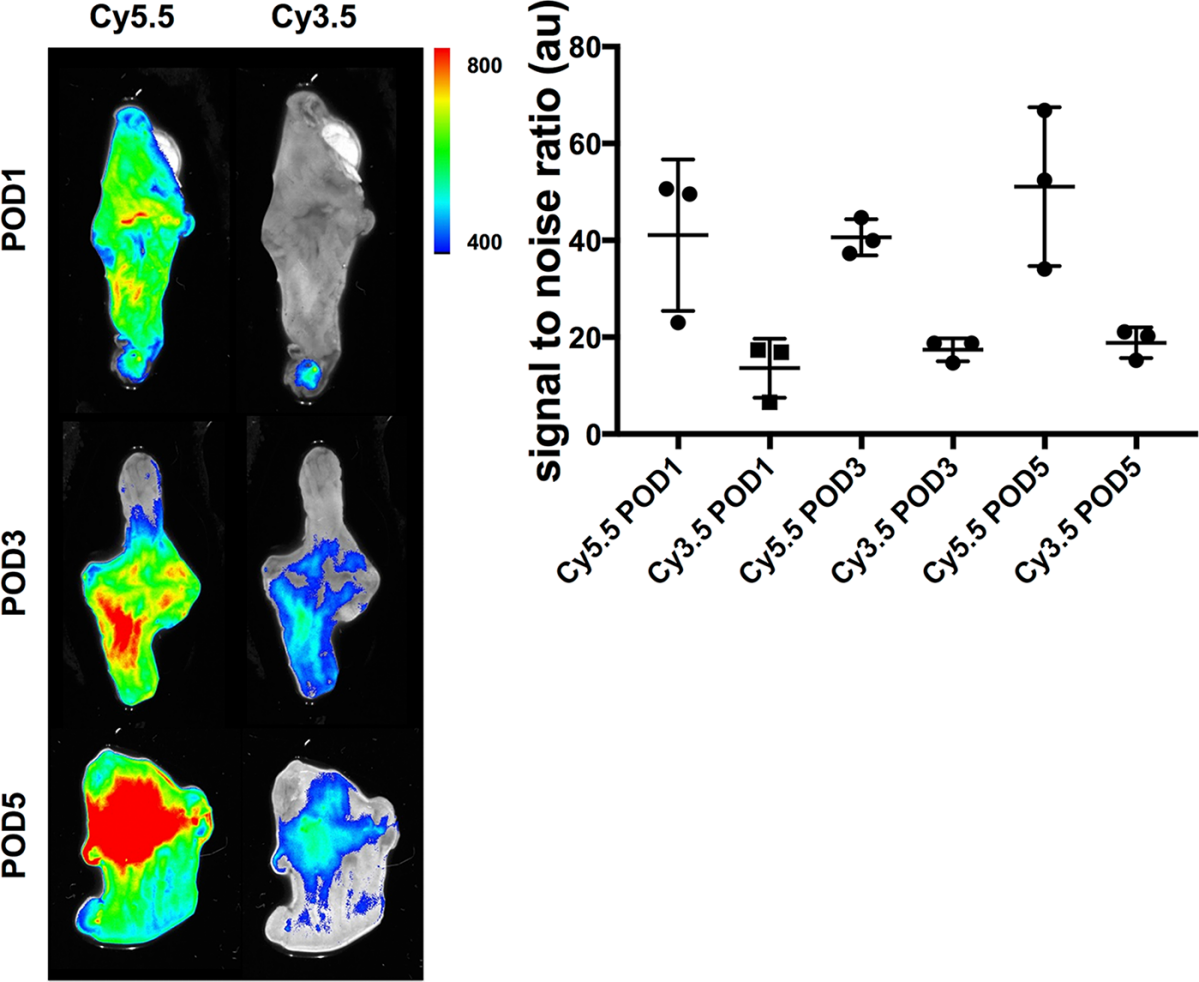
Specificity experiment to rule out unspecific binding of the tracer at the site of inflammation. **Left panel:** Ex vivo detection of the specific (Cy5.5-AF443) and unspecific (Cy3.5-Glycine) tracers using the IVIS spectrum system within the same mice was used for investigation of specificity. **Right panel:** Comparison on the different time points shows significantly higher signal to noise ratio (SNR) with the specific tracer compared to the non specific fluorophore (p = 0.0019).

The experiment to evaluate specificity of the tracer showed a significantly higher uptake in the anastomotic area of the MMP-specific tracer (Cy5.5-AF443) compared with the unspecific fluorochrome C3.5-Glycin, (p = 0.0019) thus proving specificity of the fluorescently labeled MMP inhibitor ligand within our model **(Fig 4).**

### Evaluation of fluorescence endoscopy for detection of anastomotic leakage

**Fig 5:**
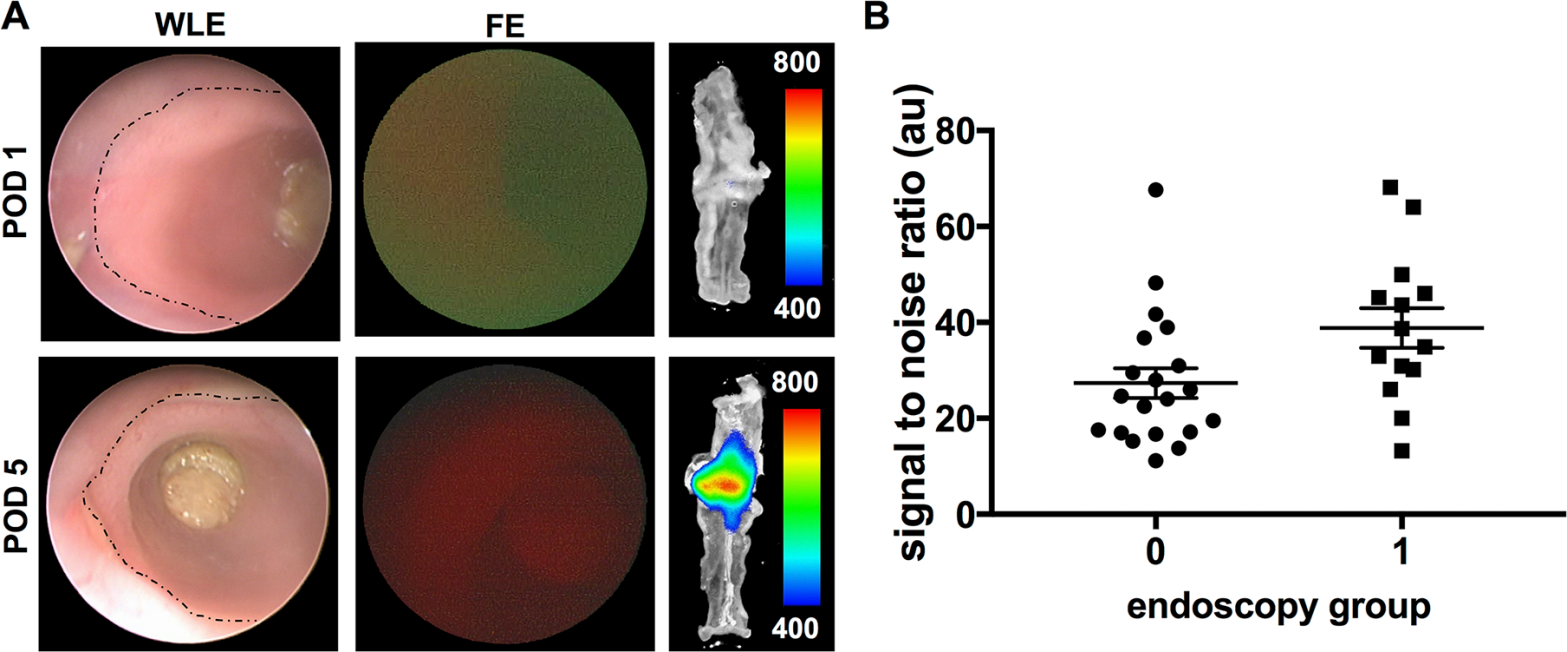
Fluorescence endoscopy can be used to detect MMP expression for analysis of anastomotic healing *in vivo*. **(A)** Fluorescence endoscopy (FE) was used for detection of the photoprobe *AF443-Cy5*.*5* in the anastomosis 24h following *i*.*v*. administration of the tracer. Areas with signs of anastomotic inflammation showed higher fluorescence signals than the rest of the anastomosis (dotted line marking the anastomotic ring). All anastomoses were analyzed and categorized into two groups as either positive (group 1) or negative (group 0). Detection of the photoprobe *in vivo* well correlated with increase in uptake of the tracer in *ex vivo* analysis (panel in the middle). **(B)** Comparison of the two groups revealed significantly higher uptake of the tracer in the group with positive signal in endoscopic analysis (p = 0.0016).

After we have shown that the tracer could be used for detection of MMP Expression in the tissue and well correlated with development of anastomotic leakage, we investigated whether the probe could also be used for MMP detection by means of fluorescence endoscopy. Therefore all anastomoses on the different days have been endoscopically evaluated and categorized into 2 groups according to the fluorescent signal (group 0: no signal, group 1: positive signal) (**Fig 5A**). For correlation whether the endoscopically detected signal would correspond to uptake of the fluorophore in the anastomosis, the *ex vivo* (optical imaging) and *in vivo* (fluorescence endoscopy) results were compared. As shown in **Fig 5B** the group with a positive signal in fluorescence endoscopy (n = 14) had a significantly higher SNR than the group without visual signal (n = 19) (SNR: 41,38 ± 4,47 vs. 24,98 ± 2,41; p = 0,0016).

We finally examined whether detection of MMP expression by fluorescence endoscopy *in vivo* could be used to identify anastomotic leakage. Thus endoscopic analysis was compared on the different postoperative days. A higher number of endoscopically positive anastomoses was detected on POD 5 (n = 6) compared to POD 1 (n = 2). Overall the number of false positive measurements was low (1 on POD1 vs. 3 on POD3 vs. 0 on POD5). Overall out of the 17 anastomoses with leakage, 10 could be detected as such while 14 out of 18 anastomoses were correctly assigned as healed, thus reflecting an overall sensitivity of 59% and specificity of 77.8%. The positive predictive value (PPV) overall was 71.4%, whereas the overall negative predictive value (NPV) was 66.6%. Within the different days there was 16.6% leakage rate on POD1 and 9 out of 10 anastomoses were correctly assigned as negative (specificity: 90%; PPV 50%; NPV: 90%). On POD3 a total of 3 out of 5 insufficiently healed anastomoses were correctly detected as such (sensitivity: 60%; specificity: 50%; PPV: 50%; NPV: 60%). Consistent with our model with increasing leakage over time, on POD5 the leakage rate was 83.3%. A total of 6 out of 10 insufficiently healed anastomoses were correctly assigned using fluorescence endoscopy (sensitivity: 60%; PPV: 100%).

## Discussion

Recent publications have shown a central role of MMP expression in anastomotic healing. Specifically expression patterns of MMP-2 and -9 have been shown to correlate with development of anastomotic leakage [4, 5, 19]. Fluorescent detection of MMPs is an established method for characterization of MMP expression in various tissues and has been used for analysis in carcinoma, arteriosclerosis and myocardial infarction models [27–29]. Recently a fluorescently labeled MMP-2/-9 specific tracer has been designed and described by Faust et al. [21] and Schwegmann et al. have subsequently validated usage of the tracer for fluorescence endoscopy to detect MMP expression in tumor development [22]. However, it has not yet been used for analysis of anastomotic healing. Taken the importance of MMP-2/-9 expression in anastomotic healing into account we were interested to evaluate fluorescently targeted endoscopic MMP detection for evaluation of MMP expression in anastomotic healing.

Endoscopy is a solid and well-established tool for analysis of inflammation and wound healing in the murine colon [30, 31]. Therefore a murine model of anastomotic leakage was chosen. For validation of the model a leakage score was calculated that combined endoscopic, macroscopic and histologic parameters of anastomotic healing. Overall an increase in anastomotic leakage rates from POD1 (17%) to POD5 (83%) was detected. Especially the endoscopic dehiscence measurements, the macroscopic adhesion score as well as the histological inflammation score showed significantly increasing leakage rates with associated inflammatory reaction on POD5 compared to POD1 (adhesion score p = 0.0002; histology p = 0.0003; dehiscence measurements p = 0.0127).

As time-points for the analysis, the postoperative days 1, 3 and 5 were chosen to investigate the early inflammatory phase of the healing process. MMP expression starts to increase within this time frame and has a central role in early organization of the extracellular matrix. Subsequent inactivation of MMP has been reported to commence on POD3 and leads to reconstruction of collagen fibers and remodeling of extracellular matrix proteins [4, 5, 19, 20]. However prolonged expression and activity of MMP can cause degradation of extracellular matrix components and therefore lead to reduction in anastomotic strength and ultimately results in anastomotic leakage [32]. On the contrary, development of anastomotic leakage can lead to continuous inflammatory stimuli that cause persistent activation of MMP, which was also reflected in our results. We have shown that mean values of the fluorescent signal of the *ex vivo* imaging showed increasing expression of MMP with increasing postoperative days reaching a maximum value at POD5. Correspondingly significantly higher signals were measured on POD5 compared to POD1 (p<0.0001). Using immunohistochemistry we have shown that MMP expression can be found within the submucosal layer of the anastomotic region with increase in expression beginning on POD3. Similar expression patterns have been described by Krarup et al. who reported elevated expression of MMP-12 in the anastomosis on POD3 compared to POD1 [20]. We have complemented the immunohistochemistry data with functional analysis of MMP activity using gelatin zymography. Altogether our data confirm previous reports that show involvement of MMPs in anastomotic leakage [5, 19]. Furthermore we have successfully evaluated a novel tool for MMP detection in the anastomosis *in vivo*. The fluorescence signals correlated well with the inflammatory parameters (*adhesion score* and *histological score*) as well as with formation of leakage. In comparison the leakage group had significantly higher MMP expression in *ex vivo* imaging than the non-leakage group (p = 0.0369). Taken together, surgical induction of anastomotic leakage led to increase of anastomotic inflammation that caused overexpression of MMP. We therefore aimed at detecting overexpression of MMPs within the anastomotic tissue as a sign of anastomotic leakage. The overarching aim of this study was to evaluate fluorescence endoscopy for detection of anastomotic leakage as a novel tool for detection of insufficiently healed anastomoses. Overall we could show that, by using the fluorescently labeled MMP Tracer *AF443-Cy 5*.*5*, fluorescence endoscopy can be used for MMP for detection of MMP in the anastomosis. Furthermore detection of MMPs well correlated with development of anastomotic leakage. In the group with the highest leakage rate on POD5 (83%), the positive predictive value increased accordingly (PPV, POD1: 50% vs. POD5: 100%). Thus in high risk anastomoses with higher likelihood of leakage, the predictive value of the method enables improvement of the diagnostic value. Overall specificity was 77.8%, on POD1 with a low leakage rate the specificity was elevated to 90%.

One of the limitations is the rather low sensitivity even in presence of a high leakage rate on POD5. A possible reason is the amount of tracer used as well as the time point chosen for analysis. Concerning the dosage, others have used varying total amounts of the tracer from 2–10 nmol [22, 26, 33], depending on the tissue and applied method of detection. In our analysis, fluorescence values measured by *ex vivo* imaging techniques were used as reference for detection of the fluorescent tracer *AF443-Cy5*.*5* and showed good uptake within in the anastomotic tissue. The tracer has been shown to exhibit high affinity and specificity to MMP-2 and -9 [21]. Although the tracer is chemically based on a barbiturate background, systemic effects are negligible due to the low dosage [22]. We have chosen a 24h interval between application of the tracer and performance of the analysis because Bettenworth et al. have detected highest uptake of the tracer in that timeframe [26]. In a different model Steingräber et al. have detected highest uptake of the tracer beginning 1h following application [33]. Extensive dosage curves were out of the scope of this project, however higher dosage as well as different time points of analysis might lead to higher sensitivity of the method. Additionally for fluorescence endoscopy a prototype system was used. One might speculate that further modification of the system, including “binning”, “real-time video enhancement” or “high resolution video endoscopy” will further improve the sensitivity of our imaging approach.

## Conclusions

We have shown that with the use of the tracer *AF443-Cy5*.*5*, fluorescent detection of MMP expression during anastomotic healing is possible. Our data supports published data showing a central role of MMP-2 and -9 in anastomotic healing. Fluorescence endoscopy promises to complement regular white light endoscopy for evaluation of anastomotic healing either for biochemical analysis in experimental settings or for clinical use. Furthermore our data has shown applicability for the method for detection of anastomotic leakage, however yet with limited sensitivity. Especially in cases with elevated risk for development of leakage, the tracers might bring important information about the inflammatory state of the anastomosis for early detection of disturbed anastomotic healing enabling early surgical or endoscopic treatment of this major complication after colorectal resections.

## Supporting information

S1 Fig Isotype controls for MMP 9 immunohistochemistry. To control for specificity of the staining isotype IGG controls were performed (right panel) on the respective postoperative days (POD). scale bar 150 μm.
(TIF)
