# Supplementary figures and images for: Assessment of MMP-2/-9 expression by fluorescence endoscopy for evaluation of anastomotic healing in a murine model of anastomotic leakage
Source: PLoS One. 2018 Mar 22;13(3):e0194249. doi: 10.1371/journal.pone.0194249 (PMC5863981; doi:10.1371/journal.pone.0194249)

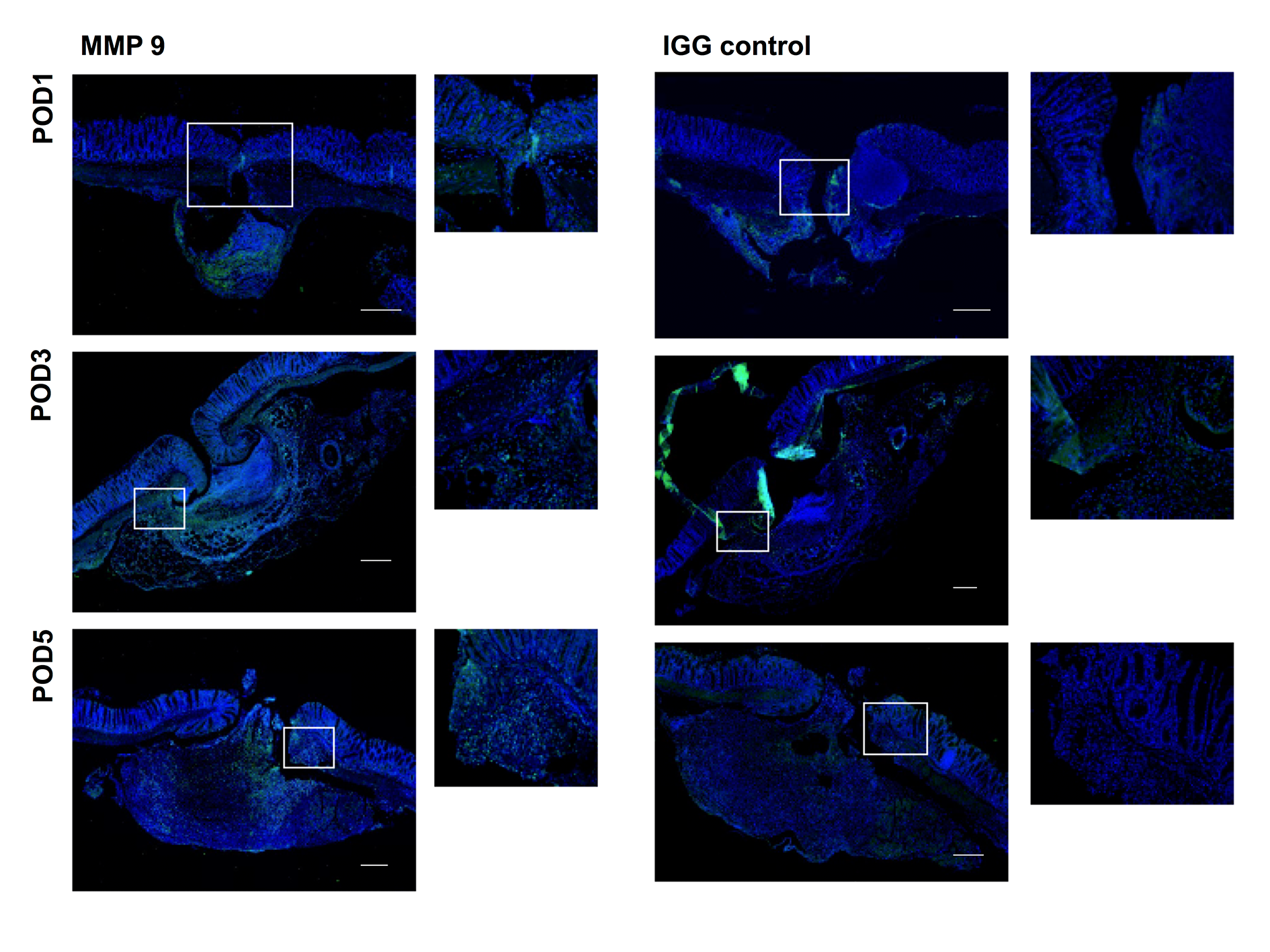

Supplement: S1 Fig — To control for specificity of the staining isotype IGG controls were performed (right panel) on the respective postoperative days (POD). scale bar 150 μm. (TIF) [file pone.0194249.s001.tif]
